# Experiences with workflows for automating data-intensive bioinformatics

**DOI:** 10.1186/s13062-015-0071-8

**Published:** 2015-08-19

**Authors:** Ola Spjuth, Erik Bongcam-Rudloff, Guillermo Carrasco Hernández, Lukas Forer, Mario Giovacchini, Roman Valls Guimera, Aleksi Kallio, Eija Korpelainen, Maciej M Kańduła, Milko Krachunov, David P Kreil, Ognyan Kulev, Paweł P. Łabaj, Samuel Lampa, Luca Pireddu, Sebastian Schönherr, Alexey Siretskiy, Dimitar Vassilev

**Affiliations:** Department of Pharmaceutical Biosciences and Science for Life Laboratory, Uppsala University, SE-75124, Uppsala, P.O. Box 591 Sweden; SLU-Global Bioinformatics Centre, Department of Animal Breeding and Genetics, Swedish University of Agricultural Sciences, Uppsala, Sweden; Science for Life Laboratory, Karolinska Institutet, SE-17121, Stockholm, P.O. Box 1031 Sweden; Division of Genetic Epidemiology, Medical University of Innsbruck, Innsbruck, 6020 Austria; CSC - IT Center for Science Ltd., FI-02101, Espoo, P.O. Box 405 Finland; Chair of Bioinformatics Research Group, Boku University, Vienna, Austria; Faculty of Mathematics and Informatics, Sofia University, Sofia, Bulgaria; CRS4 Polaris, Pula, Italy; Department of Information Technology, Uppsala University, SE-75105, Uppsala, P.O. Box 337 Sweden; AgroBioInstitute and Joint Genomic Centre, Sofia, Bulgaria

**Keywords:** Workflow, Automation, Data-intensive, High-performance computing, Big data, Reproducibility

## Abstract

High-throughput technologies, such as next-generation sequencing, have turned molecular biology into a data-intensive discipline, requiring bioinformaticians to use high-performance computing resources and carry out data management and analysis tasks on large scale. Workflow systems can be useful to simplify construction of analysis pipelines that automate tasks, support reproducibility and provide measures for fault-tolerance. However, workflow systems can incur significant development and administration overhead so bioinformatics pipelines are often still built without them. We present the experiences with workflows and workflow systems within the bioinformatics community participating in a series of hackathons and workshops of the EU COST action SeqAhead. The organizations are working on similar problems, but we have addressed them with different strategies and solutions. This fragmentation of efforts is inefficient and leads to redundant and incompatible solutions. Based on our experiences we define a set of recommendations for future systems to enable efficient yet simple bioinformatics workflow construction and execution.

**Reviewers**

This article was reviewed by Dr Andrew Clark.

## Introduction

High-throughput technologies such as next-generation sequencing (NGS) have revolutionized molecular biology and transformed it into a data-intensive discipline [1]. Bioinformaticians are nowadays required to interact with e-infrastructure consisting of high-performance computing (HPC) resources, large-scale storage, and a vibrant ecosystem of bioinformatics tools. It is common that analyses consist of multiple software tools applied in a sequential fashion on input data; and these analysis steps are usually executed on a server or a computer cluster given the significant data size and computation time requirements. Such a multi-step procedure is commonly referred to as a workflow. In order to efficiently carry out such analysis it can be beneficial to use Scientific Workflow Management Systems that can streamline the design and execution of workflows and pipelines in high-performance computing settings such as local clusters or distributed computing clouds [2].

There exist a number of workflow systems for use in bioinformatics. Taverna [3] pioneered integration of web services in bioinformatics; Galaxy [4–6] is a workflow system that has been used in sequence analysis and other bioinformatics applications; Kepler [7] and Chipster [8] are other examples of such systems that are used for next-generation sequencing and gene expression data analysis. All of the abovementioned systems have graphical user interfaces for constructing workflows and can run on HPC and cloud systems. However, experienced bioinformaticians commonly work at a lower programming level and write their workflows as custom scripts in a scripting language such as Bash, Perl or Python. For this user group, a number of lightweight workflow systems have emerged to simplify scripting and parallelizing tasks, which is particular relevant for an efficient exploitation of HPC resources, including Luigi (https://github.com/spotify/luigi), Bpipe [9], Snakemake [10] and BcBio (https://github.com/chapmanb/bcbio-nextgen). General Linux tools such as Make [11, 12] are also widely used due to their simplicity.

HPC resources in academia traditionally consist of compute clusters with Linux operating system and batch (queueing) systems for scheduling jobs. Recently, cloud computing has emerged as an additional technology offering virtualized environments and the capability to run custom virtual machine images (VMI). For workflows this opens new possibilities such as packaging entire analyses or pipelines as VMIs, which has been acknowledged in bioinformatics [13, 14]. There are also other technologies such as MapReduce [15], Hadoop [16] and Spark [17] that show great promise in bioinformatics and that might change how bioinformatics analysis can be automated.

Within the COST Action BM1006: Next Generation Sequencing Data Analysis Network (“SeqAhead”, http://www.seqahead.eu/), a series of hackathons and workshops brought together a number of scientists from different organizations, all involved in data-intensive bioinformatics analysis. This manuscript summarizes the participants’ current e-infrastructure, their experiences with workflows, lists future challenges for automating data-intensive bioinformatics analysis, and defines the criteria to enable efficient yet simple bioinformatics workflow construction and execution.

## Workflow experiences

### UPPMAX and Science for Life Laboratory, Uppsala University, Sweden

#### Overview

The Bioinformatics platform at UPPMAX and Science for Life Laboratory (SciLifeLab) provide high-performance computational resources for the national NGS community in Sweden, as well as the necessary tools and competences to enable Swedish bioinformaticians to work efficiently with HPC systems [18]. Since 2010, UPPMAX has had over 500 projects and 300 users, and as of December 2014 has 3328 compute cores and almost 7 PB of storage. On UPPMAX HPC systems, users get access to installed software, reference data, and are able to carry out data-intensive bioinformatics analyses. Applications include whole genome-, *de novo*- and exome sequencing, targeted resequencing, single nucleotide polymorphisms (SNPs) discovery, gene expression and methylation analysis.

#### Workflow experience

On our systems, most users use scripting in Bash, Perl, and Python to automate analysis. We have a security policy to not allow web servers, which has made it more difficult for us to use graphical platforms such as Galaxy. Recently, however, we have deployed a private cloud where we aim to provision images containing workflow systems like Galaxy, Chipster, and GPCR-ModSim [19], which we believe will enable us to reach a larger scientific community. We are experimenting with the workflow system Luigi on our HPC system, and CloudGene [20] on a previously established prototype Hadoop cluster in a private cloud. For automating workflow execution we use either cron jobs and an external Jenkins continuous integration instance.

Besides the workflow evaluations, considerable efforts were put on the quantitative comparison of the different approaches to solve usual bioinformatic tasks in DNA and RNA-seq experiments. In recent work we provide evidence for superior scalability for the task of mapping short reads followed by calling variants on the Hadoop-with-HDFS platform compared with the existing HPC cluster infrastructure [21]. We also developed a versatile solution [22] for the feature-counting and quality assessment tasks in RNA-seq analysis, extending the acknowledged HTSeq package [23] into the e-Science domain with Hadoop and MapReduce. We are also evaluating the Spark platform for pipelining NGS data but our initial assessment did not reveal any performance gain compare to Hadoop due to the non-iterative nature of our problems. Spark has however in our opinion a more intuitive and appealing programming environment.

#### Future challenges

It is important for UPPMAX as a national provider of HPC resources for NGS analysis to strive for efficient resource usage. With many biologists having little experience of automating bioinformatics analyses, it is important for us to provide workflow systems, examples, support, and training in order to maximize resource utilization and improve efficiency of analyses. We are noting that future pipelines will have problems running on our current HPC systems due to intensive use of shared file systems, and we will continue to evaluate and develop a future e-infrastructure where Hadoop and Spark are interesting options. There is however a challenge for traditional HPC centers like UPPMAX to adopt cloud computing and Hadoop clusters as they contrast a lot to current best practices and experiences of system administrators. The buildup of competence in these directions will be an important task.

### Chair of Bioinformatics Research Group, Boku University Vienna, Austria

#### Overview

The Chair of Bioinformatics at Boku University Vienna is a method-centric research group at the interface of computational analysis and large-scale experimental assays. Recent work includes (i) an assessment of accuracy, reproducibility, and information content of gene transcript expression profiling platforms, including RNA-Seq, microarrays, and qPCR [24]; (ii) a method benchmark in the comparison of normalization efficiency across multi-site RNA-Seq laboratories [25]; (iii) signal level models of hybridization based assays for high-density microarrays [26, 27]. These analyses require high computational power largely provided by HPC facilities like the Vienna Scientific Cluster (VSC), with the VSC-2 consisting of 1,314 nodes with 16 cores and 32 GB RAM, and the VSC-3 consisting of 2,020 nodes with 16 cores and 64 GB RAM. Large memory tasks are run on individual fat nodes with 256 GB–16 TB RAM.

#### Workflow experience

In many instances, we simply use Make [12] to run custom pipelines for both cluster and local jobs. It is a standalone tool with no setup/installation needed in most standard environments. In our experience, if a workflow system is less lightweight than Make [12] and small scripts (Perl, Bash, etc.), people will not use it when they need to ‘get something done’ even though many people know that in the long-term this is not efficient. Systems like Galaxy and Taverna provide useful platforms for the automation of routine data analysis steps as commonly found in industrial or facility settings, but are less effective for explorative and flexible analyses. In explorative work, one would like to run workflows for different configurations, and compare results. It would be helpful if there was transparent support for tagging or otherwise managing ‘alternative’ workflow runs, and outputs. Moreover, most systems lack support for the enforcement of quality control on inputs/outputs, and support for cycle control (revisions of workflows, input data, tools).

We have initially tested several systems, including, Bpipe [9], Moa [https://github.com/mfiers/Moa], Ruffus [28], and Snakemake [10]. We have since focused on exploring Snakemake due to, among other features, its make-like workflow definition, simple integration with Python, Bash code portability, ease of porting workflows to a cluster, intuitive parallelization, and ongoing active development. We are currently working on extending Snakemake with a lightweight modular system for development cycle control and policy-based specification of rules and requirements that supports an in-flow enforcement of consistency constraints. We have developed and validated a proof-of-concept prototype of the mechanism and automated the code generation of rules.

Specifically, we have used workflow systems to preprocess cancer-related data, like tumour/normal samples from the TCGA consortium [29], and to fully automate some steps of data analysis. Furthermore, we apply workflow systems in the design of high-performance microarrays for *Drosophila melanogaster* and other complex eukaryotes or to automate specialized RNA-seq analyses in fast evolving domains like single-cell profiling in stem cell research.

#### Future challenges

While Snakemake seems to be a promising tool, on its own, similar to currently available alternatives, it does not provide an exhaustive workflow system solution but instead requires external mechanisms to support critical features like revision control and management of multiple workflow instances run with varying parameter sets. We are now working to integrate Snakemake with external tools and our modular code generation system for in-flow enforcement of consistency constraints.

### CSC, Espoo, Finland

#### Overview

CSC - IT Center for Science is a government-owned computing centre in Finland that provides IT support and resources for academia, research institutes and companies. CSC provides capacity through a traditional batch oriented HPC environment, but also with a cloud platform. Major HPC environments are Cray XC40 supercomputer with 40,608 cores and HP XL230a cluster with 12,960 cores. The OpenStack based infrastructure-as-a-service (IaaS) cloud runs on the HPC cluster hardware.

As a national bioinformatics facility CSC has a large number of users, the majority of which have bio/medical background and no experience in programming. We strive to enable users to work independently by providing training and user friendly interfaces. An example of the latter is the Chipster software, developed at CSC, that provides a graphical user interface to a large suite of analysis tools [8].

#### Workflow experience

**Fig. 1:**
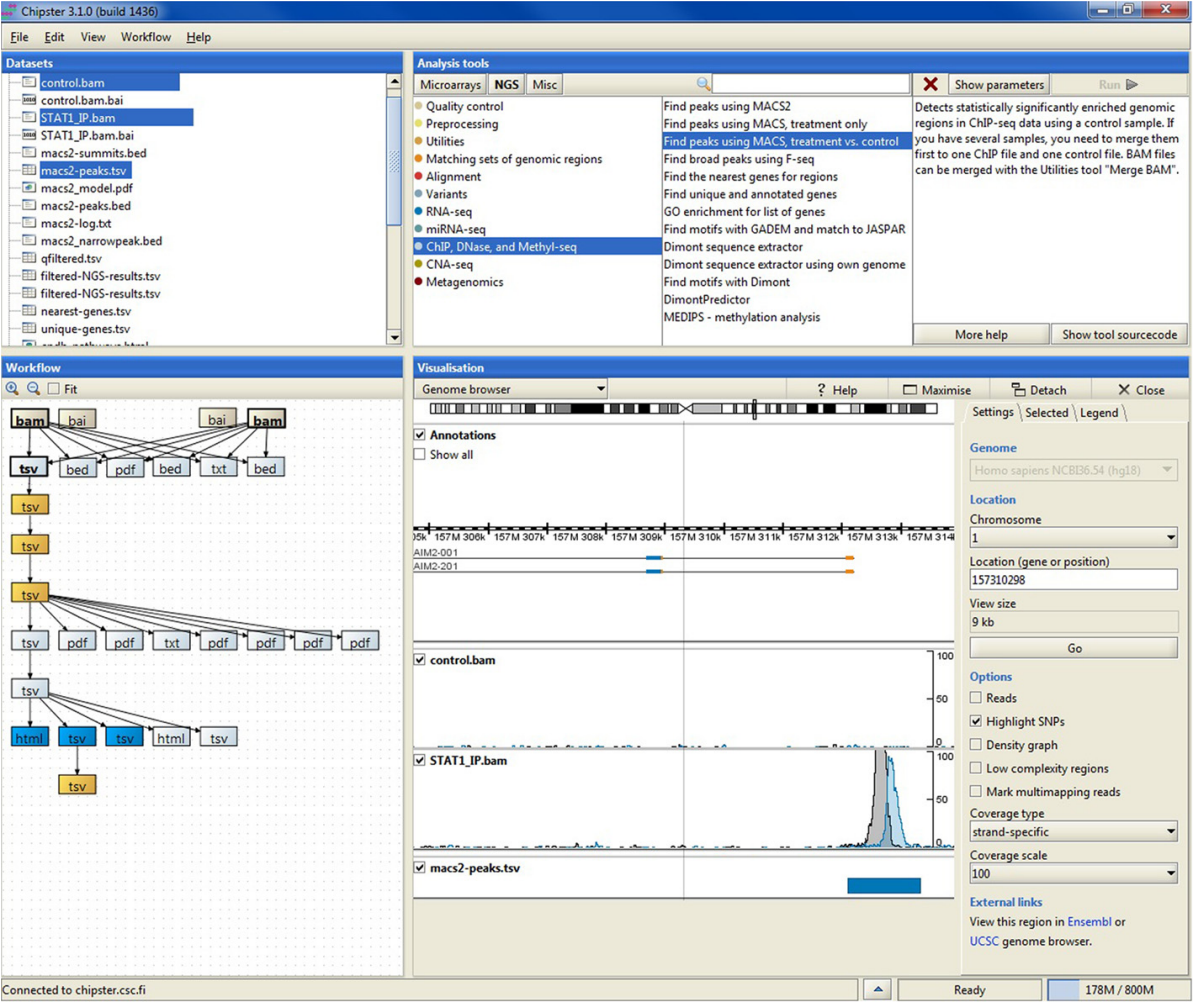
Visual representation of a user-made ChIP-seq data analysis workflow in the Chipster software. After detecting STAT1 binding regions in the genome, the user has filtered the resulting peaks for q-value, length and peak hight. S/he has then looked for common sequence motifs in the peaks and matched them against a transcription factor binding site database. S/he has also retrieved the closest genes to the peaks and performed pathway enrichment analysis for them. Finally, s/he has checked if the enriched pathways contain the STAT signaling pathway. All these downstream analysis steps can be saved as an automatic workflow, which can be shared and executed on another dataset. In addition to analysing data and building workflows, Chipster allows users to visualize data interactively. As an example, genome browser visualization is shown (bottom right panel)

Chipster enables users to create and share bioinformatics workflows. It tracks what the user does and allows him/her to save any series of analysis steps. These workflows can be exported, shared, and applied to a different dataset. Everything is tracked, including parameter settings and reference data. The result files are also automatically annotated with this information. An example of a Chipster workflow is shown in Fig. 1.

One major challenge is where to stop when recording analysis execution. We include parameters, inputs and such, but also source code for the tools. However, maintaining full reproducibility over years is impossible because the underlying tools and databases change. Our philosophy has been to maintain reproducibility to the level that is needed for workflows to be a practical tool for users. For provenance and long term archival we store enough metadata on the workflow and, most importantly, all data with their relationships. That might not be enough for one-click rerun of the pipeline several years later, but it is still enough for manual reproduction of the analysis.

Chipster users represent a wide range of research fields, ranging from medicine to agriculture and biotechnology. Therefore also the workflow functionality has to be flexible enough to cater for very different types of analysis. The typical tasks include analysis of RNA-seq data (QC, preprocessing, alignment, quantitation, differential expression analysis, filtering and pathway analysis), ChIP-seq data (QC, preprocessing, alignment, peak calling, filtering, motif discovery and pathway analysis) and exome/genome-seq data (QC, preprocessing, alignment, variant calling and filtering).

#### Future challenges

Potential future development at CSC is to provide a more technically oriented workflow engine on top of our cloud IaaS offering. We are looking into software packages that are used and developed in the cloud and big data communities as a base for our own development efforts. Workflow system would be presented with platform-as-a-service (PaaS) model. Technically capable users could program workflows that are run in the IaaS cloud, but they would not need to care about the IaaS aspects such as node provisioning and user management.

Important requirement for future workflow systems is the ability to distribute data processing workload with frameworks such as Hadoop and Spark. To this end, we have participated in development of tools that allow bioinformatics data to be efficiently processed in Hadoop: Hadoop-BAM and SeqPig [30, 31]. This work is continued by integrating Hadoop and Spark into our IaaS environment and providing easy to use interfaces for data intensive computing.

### Swedish National Genomics Infrastructure (NGI), SciLifeLab, Stockholm, Sweden

#### Overview

The Stockholm genomics core platform of the Swedish National Genomics Infrastructure (NGI) crunched over 45TBp (terabasepairs) in 2014. The current NGS instrumentation located in Stockholm includes 11 Illumina HiSeq 2500 sequencers, 3 MiSeq systems, and 3 HiSeq X sequencers, and with the coming addition of more HiSeq X instruments, the amount of data produced and processed at NGI is expected to increase dramatically in the year ahead.

#### Workflow experience

NGI in Stockholm uses bcbio-nextgen (https://github.com/chapmanb/bcbio-nextgen) and some customizations for assembling and running the analysis pipelines. For us, having support from a pipeline framework already established in other institutions has been a big plus. In our experience, home-grown bioinformatics pipeline frameworks not published or released early enough in the development process fail to gain wide adoption and momentum. As bioinformatics pipelines are inherently complex, we think it is better to share this complexity with the open source community and generalize as early as possible. Unfortunately we have not been able to keep up with fast developments upstream and periodically deploy validated instances of the pipeline.

We think that this shows the growing disconnect between traditional HPC architectures in academia and other sectors in industry:

1. *Non-community maintained software.* Such as using the ancient, hard to mantain and update “module system” (http://modules.sf.net) versus a more sustainable option such as the HomeBrew science (https://github.com/chapmanb/homebrew-cbl) system.
2. *Non-existent stable usage of cloud computing architectures*. This could enable continuous integration and delivery. Having containerized execution units coupled with good software management would increase robustness and provenance tracking on pipelines. That is, globally trackable software releases as opposed to the home-grown local module system that we now use.
3. Lack of career paths for Research Software Engineers (RSE) personnel (http://www.rse.ac.uk/who.html) that could explore new avenues and maintain points 1 and 2. In other words, lack of a “research computing” unit able to keep up and be up to date with new ways of computing.

For instance, our current HPC system does not now (and is not predicted to anytime soon) support newer deployment strategies such as continuous deployment of lightweight Docker containers (https://github.com/chapmanb/bcbio-nextgen-vm). As a result, we are actively exploring workflow frameworks and methodologies that can survive the age of HPC systems. We are investigating Piper (https://github.com/johandahlberg/piper), Snakemake, and Luigi, which seem to be more adaptable with regard to deployment strategies.

On the one hand, many pipelines incorporate a basic test suite to ensure that all moving parts work as expected. On the other hand, few of those include a benchmarking suite that can validate several bioinformatic tools and compare their performance and biological relevance. Bcbio-nextgen has put some good care in validating that the underlying biology remains sound across software versions by following up with the “Genome in a Bottle Consortium”, a gold standard for validation.

Having a continuously deployed and benchmarked pipeline allows researchers and RSEs to validate every single change in the source code, like industry does with continuous software delivery and deployment models. In this way, both source code and biology can be validated and errors spotted earlier [32]. Likewise, performance of variant callers can be continuously, closely assessed and improved quantitatively in different versions of the whole system.

#### Best practice pipeline

For a few years, bcbionextgen has been processing samples for the so called “best practice” pipeline at SciLifeLab. The typical outputs of the pipeline include:

- Quality assessment via FastQC.
- Contamination screening via fastqscreen.
- Alignment against preconfigured reference genomes and its indexes (mainly hg19).
- Variant analysis using the GATK toolkit and FreeBayes.
- Functional anotation of variants using SNPeff.
- Several RNAseq packages such as cufflinks and DEXSeq.

In practice, although the outputs are appreciated by service customers, there are many sample and project-specific details that have to be taken in consideration. This limits our ability to generalize the data that can be most useful to our scientists, but we found that at least the quality assessment and some alignment and coverage metrics are immediately useful to researchers.

#### Future challenges

Modernizing the current computing environment to more modern ways to isolate and reproduce workflows (Docker) while collaboratively managing scientific software (Homebrew Science, http://planemo.readthedocs.org/en/latest/) are big challenges that hinder reproducibility and portability. Currently, we think that systems like Piper and others are too tightly coupled with specific environments, compromising its generalization and portability.

### CRS4, Pula, Italy

#### Overview

CRS4 is a government research center with a focus on applied computing and biology. It hosts a high-throughput genotyping and sequencing facility that is directly connected to the center’s computational resources (3000 cores, 4.5 PB storage). With three Illumina HiSeq 2000 and two older Illumina Genome Analyzer IIx, it is the largest NGS platform in Italy. CRS4 directly participates in large-scale population-wide genetic studies – for instance, pertaining to autoimmune diseases and longevity [33, 34] – and provides sequencing services for external collaborators and clients. All the data produced by the sequencing laboratory undergoes some degree of processing in the computing center, spanning from quality control and packaging to reference mapping and variant calling. Over the past five years, the facility has processed more than 2000 whole-genome resequencing samples, 800 RNA-Seq samples and 200 exome sequencing samples.

#### Workflow experience

**Fig. 2:**
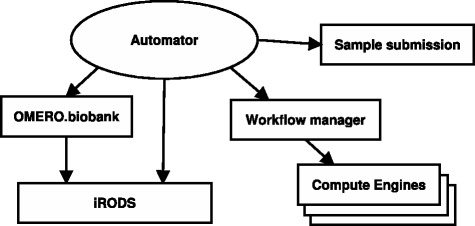
Components in CRS4’s automation system. The system has been created by linking together freely available components with some specialized software built in-house. In addition to running preliminary processing, it records operations within OMERO.biobank, thus ensuring reproducibility

**Fig. 3:**
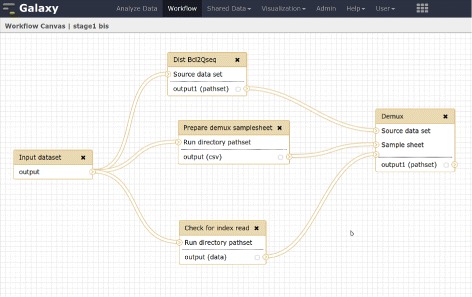
Example of a Galaxy Workflow. used at CRS4 to generates demultiplexed fastq files starting from an Illumina run directory. The BCL to qseq conversion and the demultiplexing operations are performed on a Hadoop cluster using the Seal toolkit

At CRS4 we have worked to automate the standard preliminary analysis of sequencing data to achieve high sample throughput and consistency. The processing system is summarized by the schematic diagram in Fig. 2. Our automation strategy is split in two layers. At the lower layer we are using the Galaxy platform to implement workflows for specific operations on data – e.g., demultiplexing (Fig. 3), alignment and variant calling. At a higher level, a custom daemon launches and monitors the execution of these workflows according to its configuration. When a workflow completes its operations, the daemon registers the resulting datasets in our OMERO.biobank [35] traceability framework, which allows us to keep track of which input datasets and sequence of operations were applied to produce the results (represented by serializing the galaxy history). The process effectively results in a dataset graph rooted at the original raw data.

The automation daemon also connects multiple workflow operations in sequence, when necessary; for instance, after running the demultiplexing workflow it is configured to run a sample-specific workflow to process each sample dataset. The daemon implements an event-driven model, where events are emitted in the system when something specific happens (e.g., flowcell ready, workflow finished, etc.) and the system is programmed to react to each event type with a specific action. The action may perform some housekeeping task, such as moving files to a specific location, or execute some other workflow.

To help our operation sustain a high throughput level – and to leverage CRS4’s computing cluster – we implemented some of the more time-consuming and data-intensive processing steps on the Hadoop platform [36], and proceeded to integrate these tools with Galaxy [37] to compose them with other conventional tools in our bioinformatics workflows.

In summary, our operation uses Galaxy to define complex operations (workflows) given its familiarity to biologists and bioinformaticians and its REST API, which allows is to supplement it with our own custom automation daemon. On the other hand, we have turned to Hadoop-based tools to improve our computational scalability. Finally, to ensure reproducibility we trace all our automated operations with OMERO.biobank. The entire operation is described in more detail in [35].

#### Future challenges

Future challenges vary in complexity and ambition. At a lower, perhaps simpler, level lies the need to have full reproducibility of these data analyses procedures. To a degree at CRS4 we have achieved this goal by tracing all automated operations with the combination of Galaxy and the OMERO.biobank. However, the system only works with operations that are run and monitored by our automation daemon; therefore, it cannot trace interactive, user-driven operations. In addition, our current solution introduces some complexity in managing of changes in workflows and tools versions. For these issues we currently rely on Galaxy, but its functionality in these terms is limited so alternative solutions will need to be devised or integrated.

A more ambitious challenge lies in the need to be able to efficiently deal with the steady stream of updates to model data (such as genomic references), bioinformatics tools and analysis procedures. To stay current, all acquired datasets need to be kept in line with the state-of-the-art. This is a very laborious, complex and computationally intensive task which, however, could be automated with the proper support for operating on both data and workflows computationally as first class citizens. With such functionality one could, for instance, update an alignment workflow to use the latest genome reference and automatically find all datasets that had been generated with the previous version and recompute them.

### Division of Genetic Epidemiology, Medical University of Innsbruck, Austria

#### Overview

The Medical University of Innsbruck (MUI) is one of the leading centres of medicine in Austria. Within the MUI, the Division of Genetic Epidemiology is an internationally recognized expert on lipid-associated disorders, holds cooperations with several epidemiological studies and is involved in several genome-wide association studies (GWAS) and imputation projects. An intensive cooperation with the research group Databases and Information Systems (DBIS) at the University of Innsbruck exists, developing data-intensive bioinformatics software solutions such as Cloudgene [20], HaploGrep [38] or the mtDNA-Server (http://mtdna-server.uibk.ac.at). Lately, the developed workflow system Cloudgene has been utilized as the underlying architecture for the Michigan Imputation Server, developed in cooperation with the Department of Biostatistics, University of Michigan. For in-house data analysis, a cloud approach based on a shared-nothing cluster architecture is used for data processing.

#### Workflow experience

Our institute is especially experienced in providing biomedical workflows as a service to everyone (SaaS). For example, the Michigan Imputation Server (https://imputationserver.sph.umich.edu) provides an efficient, user-friendly and free service to impute large-scale population studies using the 1000 Genomes Panel (Phase 1 and 3) or the new HRC Panel. Furthermore, the mtDNA-Server (http://mtdna-server.uibk.ac.at) enables a highly parallelized way to detect heteroplasmies and contamination within mtDNA samples.

For these time-intensive manipulation and analysis of huge datasets, we mainly focus on the application of Hadoop (hadoop.apache.org). Therefore we developed Cloudgene, a framework for the execution and tracking of Hadoop MapReduce workflows (http://cloudgene.uibk.ac.at). This graphical workflow system allows domain experts to run implemented MapReduce workflows directly from their web browsers. Cloudgene is able to combine existing MapReduce programs written in Java, approaches based on the high-level language Apache Pig, command line tools and R-based scripts to a sophisticated workflow. All used parameters and input/output data are tracked ensuring reproducibility and transparency. Final reports are created using R and RMarkdown. Within Cloudgene, workflow steps are defined in a YAML manifest file, the underlying workflow definition language (WDL) supports conditions and loops. Based on this workflow definition, Cloudgene creates user interfaces to submit MapReduce jobs graphically. Since Cloudgene supports the execution of command-line programs and bash scripts, it can also be seen as a generic workflow system. Furthermore, the architecture behind Cloudgene was developed in a way that it is compatible with existing cloud managers such as CloudMan [39, 40]. Thus, the same workflow can be executed on a local infrastructure or on private and public clouds without any adaptions [41]. This enables us to develop prototypes of new bioinformatics workflows fast and to provide them as services to other scientists.

#### Future challenges

Reproducibility of data and software is from our perspective one of the most challenging task in near future. Many publications are presenting software solutions, which are often hard to integrate into a local workflow ore impossible to use due to specific requirements on software packages. We think that cloud-based SaaS approaches applying state-of-the-art pipelines could improve the quality of current data analyses. Of course, Apache Hadoop is not applicable to all kind of problems, but its scalable and open-source nature could result in a boost within Bioinformatics. One goal of Cloudgene is therefore to improve it to an even more generic big data platform by supporting the complete Hadoop YARN architecture. This opens the door to build and execute workflows based on different computational models such as Apache Spark (http://spark.apache.org) or Apache Giraph (http://giraph.apache.org).

### Faculty of Mathematics and Informatics and ABI/Joint Genomic Centre, Sofia University, Bulgaria

#### Overview

At the Faculty of Mathematics and Informatics and Joint Genomic Center, both part of Sofia University, we do research projects that require customized workflows. This has been necessary for tasks ranging from biodiversity estimation in metagenomics, to alternative transcript detection in the wheat, maize, sorghum and arabidopsis genomes, as well as SNP discovery in wheat. For this reason, graphical or web-based workflow software designed for easy creation and maintenance of workflows does not suffice for our requirements. We started with shell scripts and then moved to standard Makefiles and as an alternative, our own Python-based bioinformatics workflow system.

#### Workflow experience

In our experience, more modern workflow systems do not always offer significant advantages. In our bioinformatics projects, the use of Makefiles alone is not enough. During tasks such as NGS assembly, alignment or variant calling, some custom data processing which cannot be implemented in shell pipelines is usually implemented in AWK, Bash or Python scripts. AWK allows compact presentation of simple data processing and is enough in surprisingly many cases. Biopython library has also proved to be very convenient for more complex handling of bioinformatics data files.

While easy to use and construct, Makefiles are often not flexible enough - their support for parallel jobs cannot take multi-threaded or multi-process jobs into account, and they do not provide any usable means to describe a recursive flow, such as progressive application of multiple alignment for large datasets. Some of the shortcomings can be overcome by using sub-Makefiles, however we thought it would be useful to develop a YAML-based workflow description system inspired by Makefiles. We apply them for the more convoluted problems, but we are hoping to make it generally applicable to simple problems as well [42].

#### Future challenges

Our aim is to build a workflow tool that is as simple as Makefiles, yet one that can make use of more complex functionality. The major challenge is making the system feature-complete and as expressive as Makefiles without sacrificing the simplicity that is inherent in the alternatives. Work is also ongoing to optimize the workflow schema and extension syntax to take maximum advantage of the YAML format. We are looking for expanding our expertise in workflow systems and improving our own tool.

## Discussion

The approaches for automating bioinformatics analysis by the organizations at the SeqAhead hackathons and workshops roughly fall into the following categories: scripting (usually in languages such as Bash, Perl, or Python), Makefiles (Make, CMake, etc.), and other workflow systems (such as Snakemake, Luigi, Galax, Taverna, and BcBio). We summarize the main advantages and disadvantages from our point of view in Table 1. One observation is that scientific workflow systems are used in two different ways. There are core workflows that are used for routine processing, are standardized, and rarely change. Then there are research workflows that a bioinformatician creates to run ad hoc analysis, explore the data and try to extract biologically relevant information. These are not and cannot be standardized and indeed the steps and parameters are chosen and modified often as the understanding of the data and the problem changes. We note that several of the organizations are not satisfied with the currently available tools and have resorted to developing in-house tools to better support their specific usage scenarios. We also observe that workflow tools developed and used in other domains, such as astronomy [43] in many cases are not widely used in bioinformatics, which may partly be due to a lack of communication between scientists of different field, yet also reflect domain specific needs.

**Table 1:** Advantages and disadvantages of different categories of automation strategies for bioinformatics

	Advantages	Disadvantages
Scripting	∙ Simple to construct	∙ Hard to hand over, manual tools integration and difficult HPC interaction
Makefile	∙ Simple to construct once you are familiar with the programming languages and the bioinformatics command-line tools involved	∙ Multithreaded programs and remote execution not handled well
	∙ Describes data flow and takes care of dependency resolution, parallel execution and caching results from previous runs	∙ Lack of recursion support
	∙ Uses code fragments in familiar scripting languages for processing of data	∙ Requires programming or shell experience
		∙ Can’t be automatically parsed and visualized
Scientific Workflow Systems	∙ More powerful features, easier to maintain and share	∙ Requires more effort

### Scripting

Shell scripts are compact and tailored for running commands in a specific order. Standard Unix/Linux systems have simple yet powerful commands for text processing, and most bioinformatics tools are available as executables that can be launched from a Shell. Other popular scripting languages like Perl or Python can also launch executables implementing bioinformatics algorithms, and additional functionality is provided by libraries that are often maintained by the community. There are some significant disadvantages to this simple approach to automation. One is that it can be tricky to ensure reproducibility of analyses; or rather, the onus is completely on the individuals who are using the script to document in some way the datasets that have been produced. Moreover, desirable advanced features such as resilience to hardware problems, the ability to re-use intermediate datasets, integration with HPC cluster resources, etc. must all be written from scratch. It can be argued that by the time such features have been integrated into the script one has effectively written a new workflow system, and thus might have been better off adopting one from the start.

However, scripting also has advantages and hence many adopters. The most important convenience is probably its simplicity and flexibility, meaning that one can very quickly achieve some degree of process automation that works, though it may not be optimal or efficient. Another important advantage is that most bioinformaticians already have scripting experience and are familiar with some scripting languages. By automating through scripts that knowledge can easily be recycled. In the authors’ experience scripting is not sufficient to provide a fault-tolerant automation for production use.

### Makefiles

The standard Unix/Linux solution for automating compilation and other tasks that follow a dependency graph are Makefiles [11]. These can serve as a simple yet effective tool to describe bioinformatics workflows, and are applicable to a wide variety of tasks. They describe dependencies between files and commands, and commands can be executed in parallel. Subsequent runs of the workflow use as much as possible of the computation files from previous runs, which serves as a basic form of caching. Drawbacks of Makefiles are their inability to describe dependencies between multiple output files per input file and a lack of support for multiple wildcards in I/O names. Moreover, the standard Unix / Linux Make tool shows limitations when encountering long running operations, and execution on heterogeneous failure-prone distributed resources. To address these issues both general purpose Make implementations, like SCons [http://www.scons.org], PGMake [44] or GXP make [45] were developed, and bioinformatrics-dedicated systems, like Makeflow [46] and Snakemake [10]. These tools try to move beyond Makefiles while retaining the simplicity of GNU Make [12].

As Makefiles grow they tend to become very complex. In the authors’ experience, Makefiles are good for simpler use cases, but have shortcomings when it comes to more complex workflows with multiple steps and branches.

### Scientific workflows

Scientific workflow system s provide an environment to interconnect components and in most cases allow for execution on distributed resources. Authors’ experiences regarding their utility vary. While all acknowledge the power and importance of scientific workflow systems to enable reproducible data analysis and simplified integration with HPC systems, in practice it turns out that many projects have started using workflow tools and frameworks but later switched back to custom scripting and Makefiles (or similar) since they discovered limitations of the systems, especially with the pressure to deliver results faster in an internationally competitive environment.

An important remaining challenge is the standardization of data flow in workflow systems. There have been several attempts to address this issue, where some are based on describing common data types via a dedicated XML schema [47, 48] or introducing ontology-based methods for managing data types [49]. No particular approach, however, has yet emerged that could substantially impact the field or find widespread acceptance in the bioinformatics community. With no central authority to dictate standards for interoperability, the community can only develop standards through collaborative efforts like the EU COST action SeqAhead.

### Key insights

- Automation on shared HPC clusters is difficult, and workflow tools can aid in achieving it.
- Full analysis reproducibility is hard, sometimes impossible to achieve. This has two reasons: i) large scale analysis very often relies on external databases that commonly are not versioned, or even if they are versioned only ’milestone’ versions are available, ii) scientific software management is on one hand inefficient in HPC clusters while on the other hand usage of the Web Services might be risky due to instability and lacking versioning. Community efforts for standardized software packaging and versioning are also lacking.
- The available log processing and provenance systems are not good enough. These would provide better reproducibility, monitoring, and analytics.
- Bioinformatics analyses are currently to a large extent file-based and there is no standardized way of passing data between applications in a workflow. This would require a transparent conversion of data formats with the resulting technical as well as semantic challenges [50]. In addition there is a necessity to check the consistency of the produced data. Although these tasks do not form a ‘research’ part of the workflow they can still constitute the majority of the workload in a typical analysis [51].
- Biological validation of workflows is typically missing. In other words, integration with a reference biological dataset, such as genome in a bottle, and accompanying test suite that validates biology across changes to external data or tools, as well as workflow revisions is, unfortunately, not a common practice today.
- Makefiles are a quick way to get the work done in a seemingly efficient manner, but the standard Make tool can become limiting when more advanced features are required. New efficient tools have been developed to address these issues (e.g. Snakemake, Bpipe).
- Scripting is common for analysis development, but we see a move to Workflow tools for data production that has strict requirements to support audits, or similar.
- Workflow systems on HPC resources have advantageous performance over cloud computing resources, but software installation is simplified on cloud systems, which can also more suitable for interactive use.

## Conclusions

Many researchers have similar problems in data-intensive bioinformatics analyses. In the authors’ experience the trend is clear in bioinformatics — workflow tools are getting increasingly more powerful, user-friendly, and hence more frequently used and appreciated for automation and creation of research pipelines. Nevertheless, the authors had to develop different ways of resolving remaining issues, which is clearly inefficient and leaves considerable room for future improvements in next-generation workflow systems.

Apart from using workflow systems the authors have developed new tools aiding workflow construction (Chipster, CloudGene, SeqPig), contributed to other workflow systems (Galaxy, Snakemake, Chipster) and analyzed frameworks such as Hadoop and Spark. Based on these experiences we have devised a set of recommendations for the next-generation automation systems for bioinformatics.

### Recommendations for future workflow systems

Bioinformatics analysis are currently to a large extent file-based, and as long as this will be the case workflow tools will continue to be important for bioinformatics automation. Even though exciting new data analytics frameworks such as Hadoop and Spark provide alternatives, with high up-front costs and the so far low uptake in the bioinformatics community we do not see a shift in paradigm within the nearest years.

The harder it is for a scientist to use a system compared to an ad-hoc hack, script, or perhaps a suboptimal stand-alone tool, the lower the widespread acceptance of a workflow system is in the wider bioinformatics and computational biology community. In general, we therefore recommend further development of lightweight and layered systems, where at least the basic functionality is easily accessed. More specifically:

- Maintain as much reproducibility as possible without sacrificing usability and simplicity of design and execution.
- Keep things simple, lightweight, easy to install and integrate with Bash and scripting languages.
- Workflows should be easily executed, with little or no change in local and distributed environments (HPC and cloud).
- Encourage attempts for further data flow standardization and data versioning as well as standardized software management.
- Put more effort into (biological) testing, validation, continuous delivery and deployment of the software. In other words, spend more effort on quality assurance.

## Reviewer’s report

### Reviewer 1: Dr Andrew Clark

The authors represent a number of impressive bioinformatics operations and share some valuable insight and experiences in this paper. Research sites engaged in similar work will no doubt relate to many of these realistic lessons learned.

The authors are right to emphasize the need for further work in the bioinformatics community on: shared community-wide standards for data, more rigor/higher quality in bioinformatics software engineering, and reproducible research/workflow methods.

I do think that hearing more real world experiences from groups fully committed to using a scientific workflow management system (WMS) would improve the concluding discussion and comparison for/against each workflow option. This perspective seems underrepresented in the views expressed. Other research fields that require data-intensive computing workflows (e.g. astronomy, physics, neuro imaging) have contributed some robust APIs and tools which are equally viable for bioinformatics applications. But such WMS options are not given much real estate in favor of quicker solutions.

Author’s response: *We agree with the reviewer and have added a note and reference to the discussion on workflow tools which from other domains which could be useful in bioinformatics.*

## Abbreviations

NGS: Next-generation sequencing
HPC: High-performance computing
VMI: Virtual machine image
PaaS: Platform-as-a-service
SaaS: Software-as-a-service
RSE: Research software engineer
GWAS: Genome-wide association study
WDL: Workflow definition language
